# Genome wide association study identifies novel potential candidate genes for bovine milk cholesterol content

**DOI:** 10.1038/s41598-018-31427-0

**Published:** 2018-09-05

**Authors:** Duy N. Do, Flavio S. Schenkel, Filippo Miglior, Xin Zhao, Eveline M. Ibeagha-Awemu

**Affiliations:** 1 Agriculture and Agri-Food Canada, Sherbrooke Research and Development Centre, Sherbrooke, QC J1M 0C8 Canada; 2 Department of Animal Science, McGill University, Ste-Anne-de-, Bellevue, QC H9X 3V9 Canada; 3 Centre for Genetic Improvement of Livestock, Department of Animal Biosciences, University of Guelph, Guelph, ON N1G 2W1 Canada; 4 Canadian Dairy Network, Guelph, ON N1K 1E5 Canada

## Abstract

This study aimed to identify single nucleotide polymorphisms (SNPs) associated with milk cholesterol (CHL) content via a genome wide association study (GWAS). Milk CHL content was determined by gas chromatography and expressed as mg of CHL in 100 g of fat (CHL_fat) or in 100 mg of milk (CHL_milk). GWAS was performed with 1,183 cows and 40,196 SNPs using a univariate linear mixed model. Two and 20 SNPs were significantly associated with CHL_fat and CHL_milk, respectively. The important regions for CHL_fat and CHL_milk were at 41.9 Mb on chromosome (BTA) 17 and 1.6–3.2 Mb on BTA 14, respectively. *DGAT1*, *PTPN1*, *INSIG1*, *HEXIM1*, *SDS*, and *HTR5A* genes, also known to be associated with human plasma CHL phenotypes, were identified as potential candidate genes for bovine milk CHL. Additional new potential candidate genes for milk CHL were *RXFP1*, *FAM198B*, *TMEM144*, *CXXC4*, *MAML2* and *CDH13*. Enrichment analyses suggested that identified candidate genes participated in cell-cell signaling processes and are key members in tight junction, focal adhesion, Notch signaling and glycerolipid metabolism pathways. Furthermore, identified transcription factors such as *PPARD*, *LXR*, and *NOTCH1* might be important in the regulation of bovine milk CHL content. The expression of several positional candidate genes (such as *DGAT1*, *INSIG1 and FAM198B*) and their correlation with milk CHL content were further confirmed with RNA sequence data from mammary gland tissues. This is the first GWAS on bovine milk CHL. The identified markers and candidate genes need further validation in a larger cohort for use in the selection of cows with desired milk CHL content.

## Introduction

Bovine milk is an important human dietary component, serving as an important delivery medium for proteins, minerals, vitamins and lipids including fatty acids and cholesterol (CHL). Milk fat is one of the principal contributors to daily dietary CHL intake for humans^1^. Milk CHL content is highly variable between species, breeds and herds and is influenced by many factors including genetics and nutrition^2,3^. Previously, we demonstrated that genetic factors contributed 10 to 18% of the total phenotypic variation in milk CHL content^4^.

High concentrations of total or low-density lipoprotein CHL (LDL-CHL) in human blood are linked to risk of cardiovascular diseases (CVD)^5–10^. Consequently, numerous genome wide association studies (GWAS) have been devoted to mapping genomic regions and variants affecting total CHL, LDL-CHL, high density lipoprotein CHL (HDL-CHL) and triglyceride^11–14^. In total, 126 GWAS have been performed on CHL related phenotypes in humans and animal model species (https://www.ebi.ac.uk/gwas/search?query=cholesterol, accessed on 09^th^ January, 2018). Although mechanisms regulating CHL have been intensively studied in humans^15–18^, few studies have been devoted to the genetics of CHL in livestock species. In cows, several gene expression/proteomics studies have reported genes with potential involvement in milk CHL concentration/metabolism^19–27^ but their actual roles and associated SNPs with CHL content in milk have not been investigated. For instance, Mani *et al*.^28^ identified ATP-binding cassette sub-family A member 1 (ABCA1) and ATP-binding cassette sub-family G member 1 (ABCG1) proteins in milk fat globule membranes and suggested their potential involvement in CHL exchange between mammary epithelial cells and alveolar milk. Using cell culture studies, Ontsouka *et al*.^21^ indicated that CHL transport in mammary epithelial cells was mediated by APOA-1/ABCA1 and ABCG1/HDL dependent pathways. Studying the response of CHL metabolism to negative energy balance induced by feed restriction, Gross *et al*.^27^ observed that CHL metabolism was influenced by nutrient and energy deficiency according to stage of lactation in dairy cows. Together, these studies^19–27^ suggest modulatory roles of cow’s genetics, physiological stage and diet on the expression of genes involved in CHL synthesis. However, the specific roles of the various genes and their sequence variants in regulating CHL synthesis and content in bovine milk have not been studied and no GWAS has been performed for milk CHL content. This study aimed to identify associated single nucleotide polymorphisms (SNPs), candidate genes and biological pathways involved in the regulation of milk CHL content via GWAS and pathway enrichment. Moreover, mRNA sequence data of mammary gland tissues from 12 cows were used to verify that the candidate genes identified by GWAS are expressed in the mammary gland.

## Results

### SNPs associated with milk cholesterol

**Figure 1:**
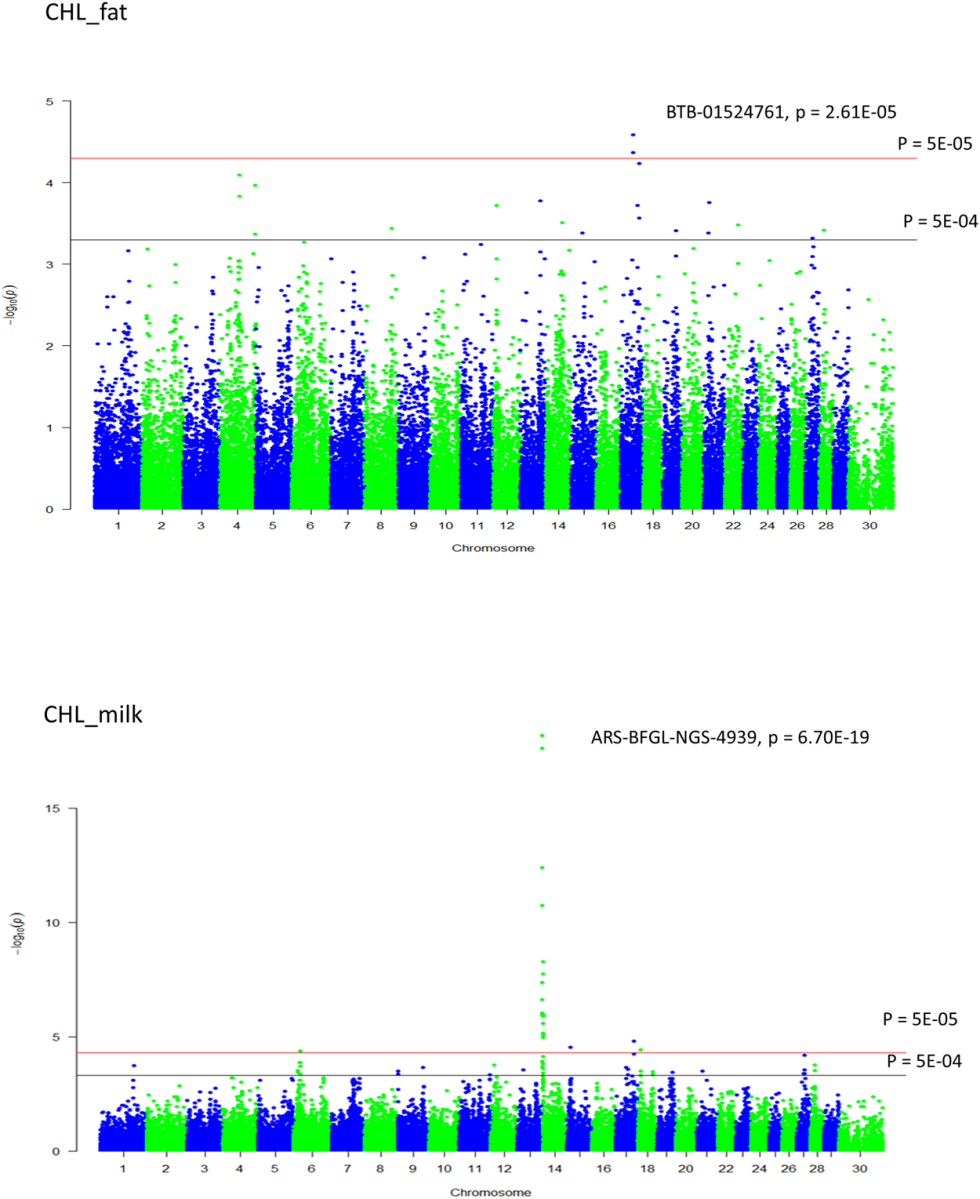
Manhattan plot of genome-wide significant (*p* < 5E-05) and suggestive (*p* < 5E-04) SNP associations for milk cholesterol content in Canadian Holstein cows. The most significant SNPs with their corresponding p-values are indicated. CHL_fat: mg of cholesterol in 100 gram of fat, CHL_milk: mg of cholesterol in 100 gram of milk.

**Figure 2:**
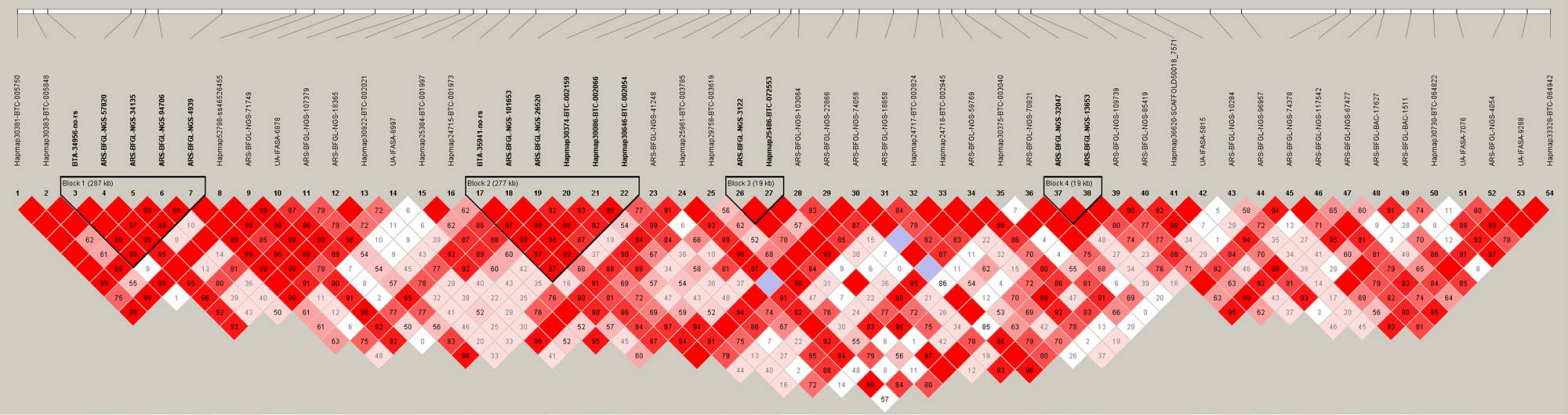
Linkage disequilibrium (LD) pattern on a 1.4–3.4 Mb region of BTA 14. LD blocks are marked with triangles; values in boxes are LD (squared correlation coefficient, r^2^) between SNP pairs; red boxes indicate LOD > 2 and D′ = 1 (LOD is the log of the likelihood odds ratio, a measure of confidence in the value of D′, where D′ is the ratio of the linkage disequilibrium coefficient D to its maximum possible).

Two and 20 SNPs were significantly associated with CHL_fat and CHL_milk, respectively at the genome wide significant threshold p < 5E-05 (Table 1, Fig. 1); while 19 and 36 SNPs (7 in common) were suggestively associated (p < 5E-04) with CHL_fat and CHL_milk, respectably (Table S1). The quantile-quantile (q-q) plot showed no systematic deviation from the diagonal (Y = X) indicating that the data were corrected for population stratification (Fig. S1). BTB-01524761 (rs42640895) and ARS-BFGL-NGS-4939 (rs109421300) were the most significantly associated SNPs with CHL_fat (p = 2.61E-05) and CHL_milk (p = 6.70E-19), respectively. Two significant SNPs for CHL_fat are located in an intergenic region of bovine chromosome (BTA) 17. The majority of significant SNPs (16 out of 20) for CHL_milk are located within a region of 1.4 to 3.3 Mb of BTA 14. Four LD blocks were detected in this region (Fig. 2) and one of the LD blocks also contained the most significant SNP (ARS-BFGL-NGS-4939 [rs109421300]) for CHL_milk. Other significant SNPs for CHL_milk are located on BTA 6, 15, 17 and 18. Several of the significant SNPs for CHL_milk are located in gene regions (seven within introns and two within exons) (Table 1). Three genes (relaxin–insulin-like family peptide receptor 1 (*RXFP1*), transmembrane protein 144 (*TMEM144)* and family with sequence similarity 198, member B (*FAM198B*)) are located in 0.5 Mb flanking regions to significant SNPs for CHL_fat. Genes including diacylglycerol O-Acyltransferase 1 (*DGAT1*), rhophilin-1 *(RHPN1)*, cysteine and histidine rich 1 *(CYHR1)*, *ENSBTAG00000003606*, vacuolar protein sorting 28 *(VPS28)*, two pore segment channel 1 *(TPCN1)*, cadherin 13 (*CDH13)*, *ENSBTAG00000045727* and MAF1 homolog, negative regulator of RNA polymerase III (*MAF1)* contained significant SNPs for CHL_milk (Table 1).

**Table 1:** Genome-wide significant SNPs for milk cholesterol content.

Trait^a^	SNP ID	BTA^b^	Position^c^	Alleles	MAF^d^	rs#	Allele_sub^e^	p-value	Consequence^f^	Gene (nearby gene)^g^
CHL_fat	Hapmap40322-BTA-100742	17	41965769	G/T	0 .340	rs41600454	11.29	4.26E-05	intergenic	(FAM198B)
CHL_fat	BTB-01524761	17	41939826	C/T	0.336	rs42640895	−11.66	2.61E-05	intergenic	*(FAM198B)*
CHL_milk	Hapmap30383-BTC-005848	14	1489496	A/G	0.423	rs109752439	0.85	1.80E-11	downstream	*ZNF34*
CHL_milk	ARS-BFGL-NGS-18858	14	2909929	A/G	0.450	rs109558046	0.71	1.76E-08	intergenic	*(ARC)*
CHL_milk	Hapmap30646-BTC-002054	14	2553525	C/T	0.356	rs110060785	0.66	1.24E-06	intergenic	*(LY6H)*
CHL_milk	ARS-BFGL-NGS-41837	6	22129886	C/T	0.212	rs110597360	0.63	4.14E-05	intergenic	*(ENSBTAG00000001751)*
CHL_milk	ARS-BFGL-NGS-18365	14	2117455	C/T	0.250	rs110892754	−0.67	2.68E-06	intergenic	*(bta_mir_2309)*
CHL_milk	Hapmap36620-SCAFFOLD50018_7571	14	3297177	C/T	0.495	rs29024688	0.58	8.37E-06	intergenic	*(TSNARE1)*
CHL_milk	Hapmap38637-BTA-88156	15	13964124	G/T	0.450	rs41596665	−0.54	2.86E-05	intergenic	*(ENSBTAG00000009511)*
CHL_milk	ARS-BFGL-NGS-4939	14	1801116	A/G	0.336	rs109421300	−1.17	6.70E-19	intron	*DGAT1*
CHL_milk	Hapmap30374-BTC-002159	14	2468020	A/G	0.490	rs109529219	0.59	7.02E-06	intron	*RHPN1*
CHL_milk	ARS-BFGL-NGS-34135	14	1675278	A/G	0.491	rs109968515	−0.66	2.34E-07	intron	*CYHR1*
CHL_milk	Hapmap30086-BTC-002066	14	2524432	A/G	0.406	rs110199901	0.77	5.14E-09	intron	*ENSBTAG00000003606*
CHL_milk	ARS-BFGL-NGS-94706	14	1696470	A/C	0.493	rs17870736	−0.70	4.27E-08	intron	*VPS28*
CHL_milk	Hapmap52830-rs29014800	17	63541690	A/G	0.403	rs29014800	−0.57	1.58E-05	intron	*TPCN1*
CHL_milk	Hapmap39330-BTA-42256	18	9797478	A/C	0.388	rs41605812	−0.54	3.63E-05	intron	*CDH13*
CHL_milk	Hapmap30922-BTC-002021	14	2138926	C/T	0.240	rs110749653	−0.64	1.12E-05	non_coding_transcript_exon	*ENSBTAG00000045727*
CHL_milk	Hapmap52798-ss46526455	14	1923292	A/G	0.396	rs41256919	−0.62	1.08E-06	synonymous	*MAF1*
CHL_milk	ARS-BFGL-NGS-57820	14	1651311	C/T	0.340	rs109146371	−1.15	2.42E-18	upstream	*FOXH1*
CHL_milk	ARS-BFGL-NGS-107379	14	2054457	A/G	0.372	rs109350371	−0.94	4.06E-13	upstream	*PLEC*
CHL_milk	BTA-35941-no-rs	14	2276443	G/T	0.498	rs41627764	−0.64	1.03E-06	upstream	*ENSBTAG00000046866*
CHL_milk	UA-IFASA-6878	14	2002873	C/T	0.419	rs41629750	−0.62	9.06E-07	upstream	*SPATC1*

^a^CHL_fat: mg of cholesterol in 100 g of fat, CHL_milk: mg of cholesterol in 100 g of milk. ^b^Bos taurus autosome. ^c^SNP position on the UMD3.1 assembly in base pairs. ^d^Minor allele frequency. ^e^Allelic substitution effect. ^f^SNP consequence obtained from Variant effect predictor (http://www.ensembl.org/Tools/VEP). ^g^Gene or nearest gene to the corresponding SNP (obtained from Ensembl gene database: http://www.ensembl.org/Bos_taurus/Info/Index.

### Gene ontology, pathways and transcription factor enrichments of positional candidate genes

**Figure 3:**
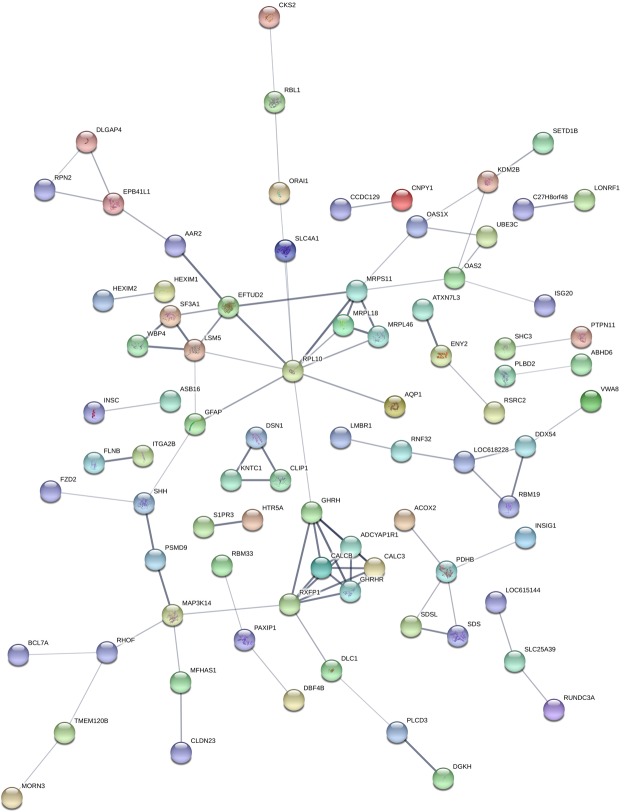
Protein-protein interaction network created using the STRING database for CHL_fat positional candidate genes. Network analysis was set at medium confidence (STRING score = 0.4). The line widths represent the level of interactions (wider lines represent stronger evidence of interactions). CHL_fat: mg of cholesterol in 100 gram of fat.

**Figure 4:**
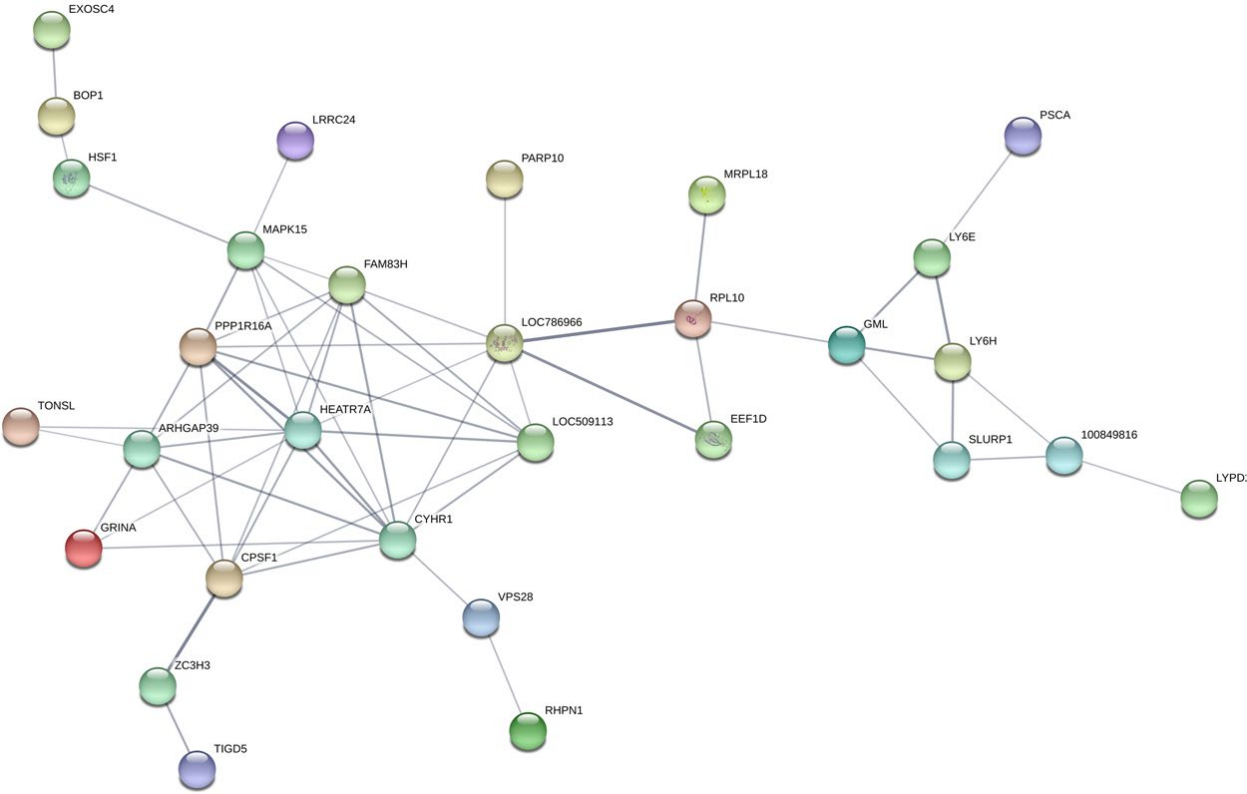
Protein-protein interaction network created using the STRING database for CHL_milk positional candidate genes. Network analysis was set at medium confidence (STRING score = 0.4). Line widths represent the level of interactions (wider lines represent stronger evidence of interactions). CHL_milk: mg of cholesterol in 100 gram of milk.

A total of 207 and 320 genes (positional candidate genes) (58 in common, Table S1) annotated at 0.5 Mb flanking regions of 21 and 56 SNPs (significant and suggestive) for CHL_fat and CHL_milk, respectively (Table S1), were used as input for GO and pathways enrichment. A total of 59 and 112 GO terms were enriched for CHL_fat and CHL_milk positional candidate genes, respectively (Table S2). For CHL_fat, negative regulation of cyclin-dependent protein kinase activity (p = 0.001), basolateral plasma membrane (p = 0.007) and cyclin-dependent protein kinase regulator activity (p = 1.10E-04) were the most significant biological processes, cellular component and molecular function GO terms, respectively, enriched for positional candidate genes (Table 2). Meanwhile, cardiac muscle tissue development (p = 1.10E-04), anchored to membrane (p = 0.001) and interleukin-2 receptor binding (p = 8.60E-05) were the most significant biological processes, cellular component and molecular function GO  terms, respectively, enriched for CHL_milk positional candidate genes (Table 3). In addition, 5 KEGG pathways (neuroactive ligand-receptor interaction, focal adhesion, leukocyte transendothelial migration, tight junction and basal cell carcinoma) and 2 (glycerolipid metabolism and Notch signaling) were enriched for CHL_fat and CHL_milk positional candidate genes, respectively (Tables 2 and 3). The potential interactions between the positional candidate genes for CHL_fat and CHL_milk are shown in Figs 3 and 4, respectively. *PRL10*, *GHRH*, *CALCB* and *RXFP1* interacted highly with other genes for CHL_fat (Fig. 3) while *MAPK15*, *FAM83H*, *ARHGAP39*, *HEATR7A*, *CYHR1* and *CPSF1* were among highly interacting genes in the CHL_milk protein interaction network (Fig. 4). Moreover, a total of 20 and 16 transcription factors were enriched for positional candidate genes for CHL_fat and CHL_milk, respectively (Table 4). The most enriched transcription factors for CHL_fat were *CREB1* (p = 0.002), *PPARD* (p = 0.004) and *CEBPB* (p = 0.005) and for CHL_milk were *LXR* (p = 1E-11), *DACH1* (p = 1E-07) and *SMC4* (p = 1.19E-07).

**Table 2:** Gene ontology and pathways enriched for positional candidate genes of CHL_fat^a^.

Category^b^	Names	Number of genes	p-value
GO_BP	Negative regulation of cyclin-dependent protein kinase activity	2	0.001
GO_BP	Cell-cell signaling	5	0.001
GO_BP	Cell communication	6	0.004
GO_BP	Regulation of cyclin-dependent protein kinase activity	2	0.006
GO_BP	Regulation of nervous system development	3	0.007
GO_BP	Organic acid catabolic process	3	0.008
GO_BP	Carboxylic acid catabolic process	3	0.008
GO_BP	Regulation of adenylate cyclase activity	2	0.009
GO_BP	G-protein signaling, coupled to cAMP nucleotide second messenger	2	0.009
GO_BP	G-protein signaling, coupled to cyclic nucleotide second messenger	2	0.009
GO_BP	cAMP-mediated signaling	2	0.010
GO_CC	Basolateral plasma membrane	3	0.007
GO_MF	Cyclin-dependent protein kinase regulator activity	3	1.10E-04
GO_MF	snRNA binding	2	4.80E-04
GO_MF	Cyclin-dependent protein kinase inhibitor activity	2	0.001
GO_MF	Protein serine/threonine kinase inhibitor activity	2	0.003
GO_MF	Protein kinase regulator activity	3	0.003
GO_MF	Kinase regulator activity	3	0.005
GO_MF	Protein kinase inhibitor activity	2	0.006
GO_MF	Kinase inhibitor activity	2	0.008
KEGG	Neuroactive ligand-receptor interaction	5	0.015
KEGG	Focal adhesion	4	0.026
KEGG	Leukocyte transendothelial migration	3	0.032
KEGG	Tight junction	3	0.040
KEGG	Basal cell carcinoma	2	0.043

^a^CHL_fat: mg of cholesterol in 100 g of fat. Only gene ontologies with p-values < 0.01 are shown.

^b^GO_BP: Biological processes gene ontology term, GO_CC: Cellular component gene ontology term and GO_MF: Molecular function gene ontology term.

**Table 3:** Gene ontology and pathways enriched for potential candidate genes of CHL_milk^a^.

Category^b^	Names	Number of genes	p-value
GO_BP	Cardiac muscle tissue development	4	1.00E-04
GO_BP	Positive regulation of cell-matrix adhesion	2	4.30E-04
GO_BP	Heart development	5	0.001
GO_BP	Negative regulation of protein ubiquitination	2	0.002
GO_BP	Striated muscle tissue development	4	0.002
GO_BP	Muscle tissue development	4	0.003
GO_BP	Ribosome biogenesis	4	0.003
GO_BP	Ventricular cardiac muscle morphogenesis	2	0.003
GO_BP	Regulation of cell-matrix adhesion	2	0.005
GO_BP	Cardiac muscle cell differentiation	2	0.005
GO_BP	Negative regulation of translation	2	0.005
GO_BP	Cardiac muscle tissue morphogenesis	2	0.005
GO_BP	Muscle tissue morphogenesis	2	0.005
GO_BP	Cardiac cell differentiation	2	0.005
GO_BP	Ribonucleoprotein complex biogenesis	4	0.006
GO_BP	Nuclear-transcribed mRNA catabolic process, nonsense-mediated decay	2	0.006
GO_BP	Muscle organ development	4	0.006
GO_BP	rRNA processing	3	0.008
GO_BP	Negative regulation of cellular protein metabolic process	3	0.008
GO_BP	rRNA metabolic process	3	0.008
GO_BP	Regulation of protein ubiquitination	2	0.008
GO_BP	Negative regulation of protein metabolic process	3	0.009
GO_BP	Regulation of macromolecule metabolic process	21	0.009
GO_BP	Notch signaling pathway	2	0.009
GO_BP	Regulation of cell proliferation	7	0.009
GO_BP	Negative regulation of cellular process	11	0.009
GO_BP	Anatomical structure formation involved in morphogenesis	5	0.010
GO_CC	Anchored to membrane	5	0.001
GO_CC	Intracellular	66	0.006
GO_MF	Interleukin-2 receptor binding	2	8.60E-05
GO_MF	ATP-dependent helicase activity	5	2.50E-04
GO_MF	Purine NTP-dependent helicase activity	5	2.50E-04
GO_MF	Nucleic acid binding	31	0.002
GO_MF	Helicase activity	5	0.002
GO_MF	ATPase activity, coupled	6	0.006
GO_MF	3′–5′ exonuclease activity	2	0.006
KEGG	Glycerolipid metabolism	2	0.043
KEGG	Notch signaling pathway	2	0.045

^a^CHL_milk: mg of cholesterol in 100 g of milk. Only gene ontologies with p-values < 0.01 are shown.

^b^GO_BP: Biological processes gene ontology term, GO_CC: Cellular component gene ontology term and GO_MF: Molecular function gene ontology term.

**Table 4:** Significantly enriched transcription factors for positional candidate genes for CHL_fat and CHL_milk.

Trait^a^	Transcription factor	Overlap	p-value
CHL_fat	*CREB1*	40/3057	0.002
CHL_fat	*PPARD*	11/516	0.004
CHL_fat	*CEBPB*	9/382	0.005
CHL_fat	*MYC*	14/797	0.006
CHL_fat	*GRHL2*	16/1000	0.009
CHL_fat	*CIITA*	9/459	0.014
CHL_fat	*CLOCK*	8/407	0.020
CHL_fat	*NANOG*	13/840	0.022
CHL_fat	*FOXP3*	19/1404	0.023
CHL_fat	*E2A*	25/2000	0.023
CHL_fat	*SMAD4*	25/2000	0.023
CHL_fat	*FOXA1*	25/2000	0.023
CHL_fat	*TFAP2A*	24/1904	0.024
CHL_fat	*TAL1*	23/1875	0.035
CHL_fat	*MITF*	57/5578	0.036
CHL_fat	*ATF3*	26/2189	0.036
CHL_fat	*EST1*	14/1001	0.038
CHL_fat	*CTCF*	24/2000	0.039
CHL_fat	*EOMES*	13/932	0.045
CHL_fat	*NFIB*	9/573	0.048
CHL_milk	*LXR*	60/2000	1.00E-11
CHL_milk	DACH1	46/1698	1.00E-07
CHL_milk	*SMC4*	51/2000	1.19E-07
CHL_milk	*BCL6*	39/2000	0.001
CHL_milk	*P68*	39/2000	0.001
CHL_milk	*ZNF274*	11/327	0.002
CHL_milk	*P300*	38/2000	0.003
CHL_milk	*EZH2*	20/935	0.008
CHL_milk	*EGR1*	91/6207	0.010
CHL_milk	*KDM2B*	35/2000	0.013
CHL_milk	*MYCN*	7/234	0.022
CHL_milk	*NOTCH1*	7/245	0.028
CHL_milk	*ERG*	8/321	0.039
CHL_milk	*PRDM5*	19/1029	0.039
CHL_milk	*FOXO3*	14/695	0.039
CHL_milk	*EWS-FLI1*	12/574	0.043

^a^CHL_fat: mg of cholesterol in 100 g of fat, CHL_milk: mg of cholesterol in 100 g of milk.

### Pearson correlation of candidate gene expression (read counts) in mammary gland tissues with milk cholesterol content

Examination of RNA sequence data (read counts) of mammary gland tissues from 12 cows at mid lactation (day 120–180) indicated that among 207 positional candidate genes for CHL_fat, 35 genes were not expressed, 25 genes were very lowly expressed (each with total read counts <10), while 12 genes (*TMEM120B*, *INSIG1*, *FLNB*, *RPN2*, *RASAL1*, *ARF4*, *MYL9*, *GRN*, *ORAI1*, *PLBD2*, *AQP1* and *RSRC2*) were highly expressed (each with total read counts >10,000) (Table S4a,b). Out of 320 genes for CHL_milk, 70 genes were not expressed, 36 genes were lowly expressed (each with total read counts <10), while 19 genes were highly expressed (each with total read counts >10,000) (Table S4a,c). *LGB*, *RPL8*, *RPS19*, *EEF1D*, *ITGB1* and *HNRNPF* were the most highly expressed genes among the CHL_milk positional candidate genes. Moreover, the expression of 45 out of 207 CHL_fat and 72 out of 320 CHL_milk positional candidate genes was significantly correlated with CHL_fat and CHL_milk, respectively (Tables 5 and 6). The expression of genes including *EPB41L1*, *DET1*, *DTX1*, *ABHD6*, *RSRC2*, *ITGA2B*, *MLXIP*, *KCTD6* and *DLGAP4* was strongly and significantly correlated (|cor| > 0.8 and p < 0.01) to CHL_fat (Table 5). Moreover, the expressions of 28 genes were strongly and significantly correlated (|cor| > 0.8 and p < 0.01) with CHL_milk (Table 6) including *ENSBTAG00000048096* and *TONSL*, as the two most significantly correlated (|cor| > 0.9 and p < 0.001) to CHL_milk.

**Table 5:** Positional candidate genes for milk cholesterol which are expressed in mammary gland tissues and also significantly correlated to cholesterol concentration in milk fat (CHL_fat)^a^ of the same cows.

Ensembl Gene^b^	Gene symbol	Total read counts	cor_CHL_fat^c^	p_cor_CHL_fat
*ENSBTAG00000001640*	*EPB41L1*	3115	−0.893	0.001
***ENSBTAG00000000967***	***DET1***	974	−0.892	0.001
***ENSBTAG00000016738***	***DTX1***	1331	−0.830	0.006
*ENSBTAG00000016615*	*ABHD6*	620	−0.828	0.006
*ENSBTAG00000006118*	*RSRC2*	11237	−0.827	0.006
*ENSBTAG00000008165*	*ITGA2B*	1190	−0.822	0.007
*ENSBTAG00000004189*	*MLXIP*	1609	−0.815	0.007
*ENSBTAG00000022656*	*KCTD6*	2746	−0.804	0.009
*ENSBTAG00000001741*	*DLGAP4*	2049	−0.802	0.009
*ENSBTAG00000017505*	*PAXIP1*	1250	−0.790	0.011
*ENSBTAG00000019989*	*PXK*	1309	−0.767	0.016
*ENSBTAG00000020590*	*FZD2*	242	−0.767	0.016
*ENSBTAG00000007387*	*ENY2*	3025	−0.766	0.016
***ENSBTAG00000016637***	***WBP4***	2544	−0.763	0.017
*ENSBTAG00000008025*	*UBE3C*	4692	−0.763	0.017
***ENSBTAG00000037527***	***OAS1Z***	509	−0.763	0.017
***ENSBTAG00000048096***	***ENSBTAG00000048096***	4	0.760	0.017
*ENSBTAG00000006114*	*ZCCHC8*	3333	−0.758	0.018
***ENSBTAG00000001133***	***VWA8***	1968	−0.757	0.018
*ENSBTAG00000038316*	*GPATCH8*	4564	−0.753	0.019
***ENSBTAG00000010694***	***BICC1***	545	−0.750	0.020
***ENSBTAG00000047729***	***ENSBTAG00000047729***	20	0.749	0.020
*ENSBTAG00000021669*	*SOGA1*	456	−0.745	0.021
*ENSBTAG00000020802*	*ENSBTAG00000020802*	921	−0.742	0.022
*ENSBTAG00000011447*	*FAM171A2*	196	−0.741	0.022
*ENSBTAG00000007084*	*MAP3K14*	1583	−0.720	0.029
*ENSBTAG00000021164*	*SLMAP*	6735	−0.717	0.030
*ENSBTAG00000016435*	*NOM1*	2232	−0.714	0.031
***ENSBTAG00000017069***	***FAM198B***	1089	−0.709	0.033
*ENSBTAG00000006051*	*NMT1*	4740	−0.704	0.034
*ENSBTAG00000030817*	*LMBR1*	3270	−0.704	0.034
*ENSBTAG00000013526*	*EFTUD2*	6186	−0.703	0.035
***ENSBTAG00000039861***	***OAS1Y***	1418	−0.695	0.038
***ENSBTAG00000015913***	***MFHAS1***	386	−0.694	0.038
*ENSBTAG00000011473*	*MYL9*	20172	−0.687	0.041
*ENSBTAG00000004199*	*DIABLO*	2763	−0.683	0.042
*ENSBTAG00000019463*	*SLC25A39*	9582	−0.683	0.042
*ENSBTAG00000000357*	*ENSBTAG00000000357*	4120	−0.683	0.042
*ENSBTAG00000019987*	*RPP14*	4821	−0.681	0.043
***ENSBTAG00000007051***	***CLDN23***	62	−0.679	0.044
*ENSBTAG00000015541*	*DLC1*	2635	−0.679	0.044
*ENSBTAG00000018433*	*DENND6A*	1496	−0.679	0.044
*ENSBTAG00000018823*	*GRN*	19107	−0.677	0.045
*ENSBTAG00000047599*	*GHRHR*	21	−0.671	0.048
*ENSBTAG00000022004*	*FLNB*	34529	−0.669	0.049

^a^CHL_fat: mg of cholesterol in 100 g of fat, CHL_milk: mg of cholesterol in 100 g of milk.

^b^Genes in bold face are also positional candidate genes for CHL_milk.

^c^Pearson correlation coefficient.

**Table 6:** Positional candidate genes for milk cholesterol which are expressed in mammary gland tissues and also significantly correlated to cholesterol concentration in milk (CHL_milk)^a^ of the same cows.

Ensembl Gene^b^	Gene symbol	Total read counts	cor_CHL_milk^c^	p_cor_CHL_milk
***ENSBTAG00000048096***	***ENSBTAG00000048096***	4	0.933	2.39E-04
*ENSBTAG00000007749*	*TONSL*	634	−0.923	3.84E-04
*ENSBTAG00000015910*	*ITGB1*	44254	−0.897	0.001
***ENSBTAG00000000967***	***DET1***	974	−0.897	0.001
*ENSBTAG00000024889*	*HSBP1*	7826	−0.893	0.001
*ENSBTAG00000018456*	*ZNF7*	1524	−0.892	0.001
*ENSBTAG00000039328*	*PURG*	47	−0.876	0.002
*ENSBTAG00000005691*	*FGF2*	2308	−0.871	0.002
*ENSBTAG00000013125*	*PLAUR*	332	−0.868	0.002
*ENSBTAG00000045791*	*ZNF623*	845	−0.863	0.003
*ENSBTAG00000018975*	*KCNT1*	555	−0.857	0.003
*ENSBTAG00000002883*	*RPTOR*	2659	−0.847	0.004
*ENSBTAG00000013439*	*ARHGEF26*	2619	−0.839	0.005
*ENSBTAG00000006132*	*DENND3*	4706	−0.835	0.005
*ENSBTAG00000018912*	*ARHGEF1*	10394	−0.829	0.006
*ENSBTAG00000030939*	*ZNF575*	287	−0.828	0.006
*ENSBTAG00000014607*	*EXOSC4*	988	−0.821	0.007
*ENSBTAG00000001262*	*IRGQ*	498	−0.819	0.007
*ENSBTAG00000019864*	*MAPK15*	751	−0.814	0.008
*ENSBTAG00000039851*	*UBAC1*	6064	−0.813	0.008
*ENSBTAG00000012796*	*ZNF428*	465	−0.811	0.008
*ENSBTAG00000016268*	*XRCC1*	2290	−0.809	0.008
*ENSBTAG00000000312*	*GRINA*	6104	−0.808	0.008
*ENSBTAG00000021472*	*ZC3H3*	1032	−0.807	0.009
*ENSBTAG00000004092*	*AK8*	372	−0.805	0.009
*ENSBTAG00000004969*	*LRRC14*	1730	−0.805	0.009
***ENSBTAG00000016738***	***DTX1***	1331	−0.802	0.009
*ENSBTAG00000011815*	*SMG9*	2101	−0.801	0.009
*ENSBTAG00000015267*	*SGSH*	2811	−0.799	0.010
***ENSBTAG00000031824***	***RBM19***	2179	−0.799	0.010
*ENSBTAG00000026356*	*DGAT1*	4493	−0.794	0.011
*ENSBTAG00000013283*	*PRR19*	309	−0.792	0.011
*ENSBTAG00000020754*	*ZNF526*	1161	−0.792	0.011
*ENSBTAG00000004173*	*UBXN8*	2079	−0.790	0.011
*ENSBTAG00000008853*	*HNRNPF*	35493	−0.786	0.012
*ENSBTAG00000011064*	*ADCK5*	3161	−0.777	0.014
*ENSBTAG00000003606*	*ZNF16*	1067	−0.773	0.015
*ENSBTAG00000006581*	*CCDC82*	1850	−0.759	0.018
*ENSBTAG00000016810*	*PYCRL*	6075	−0.757	0.018
*ENSBTAG00000010606*	*PPP1R3B*	607	−0.757	0.018
***ENSBTAG00000010947***	***PHYHIPL***	6186	−0.754	0.019
*ENSBTAG00000020236*	*NECAB2*	163	−0.753	0.019
*ENSBTAG00000026320*	*VPS28*	6020	−0.752	0.019
*ENSBTAG00000020756*	*GSK3A*	5533	−0.751	0.020
***ENSBTAG00000038494***	***ENSBTAG00000038494***	330	−0.743	0.022
*ENSBTAG00000001826*	*SASH1*	2268	−0.739	0.023
*ENSBTAG00000019785*	*CIC*	6558	−0.735	0.024
***ENSBTAG00000011102***	***TPCN1***	6605	−0.727	0.026
*ENSBTAG00000019866*	*NRP1*	7819	−0.727	0.027
*ENSBTAG00000018455*	*COMMD5*	2136	−0.727	0.027
*ENSBTAG00000002976*	*CD177*	44	−0.727	0.027
*ENSBTAG00000011963*	*RPS19*	57636	−0.724	0.028
*ENSBTAG00000007115*	*GSR*	2239	−0.724	0.028
***ENSBTAG00000047729***	***ENSBTAG00000047729***	20	0.721	0.028
*ENSBTAG00000033727*	*RBPMS*	1632	−0.718	0.029
*ENSBTAG00000003530*	*DDX31*	16551	−0.711	0.032
***ENSBTAG00000011937***	***RITA1***	1067	−0.710	0.032
*ENSBTAG00000009677*	*PARP10*	3006	−0.702	0.035
*ENSBTAG00000014458*	*MROH1*	8527	−0.701	0.035
*ENSBTAG00000035254*	*CYHR1*	4420	−0.697	0.037
***ENSBTAG00000019040***	***PLBD2***	14432	−0.697	0.037
*ENSBTAG00000014610*	*GPAA1*	13022	−0.696	0.037
*ENSBTAG00000005761*	*DEDD2*	2653	−0.695	0.038
*ENSBTAG00000012691*	*GTF2E2*	4154	−0.693	0.038
*ENSBTAG00000007834*	*PPP1R16A*	1451	−0.692	0.039
*ENSBTAG00000001260*	*PINLYP*	7	−0.686	0.041
*ENSBTAG00000040086*	*SLC38A8*	7	−0.686	0.041
*ENSBTAG00000012235*	*SHARPIN*	1729	−0.686	0.042
*ENSBTAG00000011103*	*SLC8B1*	4800	−0.679	0.044
*ENSBTAG00000006008*	*CAMSAP1*	2406	−0.675	0.046
*ENSBTAG00000009245*	*PPP2CB*	12515	−0.674	0.047
*ENSBTAG00000014642*	*NAPRT*	17674	−0.668	0.049

^a^CHL_fat: mg of cholesterol in 100 g of fat, CHL_milk: mg of cholesterol in 100 g of milk.

^b^Genes in bold face are also positional candidate genes for CHL_fat.

^c^Pearson correlation coefficient.

## Discussion

It is known that most cow milk CHL (about 80%) is derived from blood whereas a small portion (about 20%) is derived through local synthesis in the mammary gland^29^. Therefore, the regulation of milk CHL content may require complex mechanisms and the involvement of many genes and pathways. Recently, we reported heritability estimates for CHL_fat (0.09) and CHL_milk (0.18) suggesting that genetics contributes a proportion of the total phenotypic variances in milk CHL content^4^.

More SNPs (20) were significantly associated with CHL_milk as compared to two for CHL_fat at the genome wide significant threshold (p < 5E-05). Furthermore, 36 and 19 SNPs including 7 in common were suggestively associated (p < 5E-04) with CHL_milk and CHL_fat, respectively. In fact, 58 genes are located in 0.5 Mb flanking regions of 7 suggestively (p < 5E-04) associated SNPs (ARS-BFGL-NGS-110646 [rs109154988], ARS-USMARC-Parent-DQ786763-rs29020472 [rs29020472], BTB-01524761 [rs42640895], BTB-01712106 [rs42829960], Hapmap40322-BTA-100742 [rs41600454], Hapmap43002-BTA-63541 [rs41586803], and Hapmap52830-rs29014800 [rs29014800]) for CHL_milk and CHL_fat. Some of the genes have been reported to have potential roles in CHL metabolism such as protein tyrosine phosphatase 1β *(PTPN1)*, diacylglycerol kinase eta (*DGKH)* and serine dehydratase (*SDS*). *PTPN1* is an important gene for plasma total and HDL-CHL^30–33^ while *DGKH* encodes an enzyme responsible for the recycling and degradation of diacylglycerol, known as important for CHL efflux from adipose cells^34^. SDS gene on the other hand is known to contain a susceptibility loci for low HDL-CHL levels^35^. The most important QTL region for CHL_fat at 41.9 Mb of BTA 17 contained two significant SNPs (Hapmap40322-BTA-100742 [rs41600454] and BTB-01524761 [rs42640895]) for the trait. Relaxin–insulin-like family peptide receptor 1 (*RXFP1*), transmembrane protein 144 (*TMEM144)* and family with sequence similarity 198, member B (*FAM198B*) genes are positional candidate genes for CHL_fat, however, none of them has been reported to have a direct role in the regulation of CHL metabolism. *RXFP1*, one of four relaxin receptors, is known to play a role in signal transduction between extracellular/intracellular domains^36^. The activation of RXFP1 receptor stimulates the phosphorylation of mitogen-activated protein kinases such as ERK1/2^36^. In fact, the phosphorylation of ERK1/2 is important for the regulation of CHL efflux^37^. *RXFP1* is also among genes with more levels of interactions with other CHL-fat candidate genes, as shown by the interaction network (Fig. 3). However, *RXFP1* was very lowly expressed in mammary gland tissues (Table S4) so its involvement with CHL_fat concentration might be through its activities in other tissues. The involvement of *FAM198B* and *TMEM144* genes in CHL metabolism might be via their roles in the membrane, since *TMEM144* is a carbohydrate transmembrane transporter while *FAM198B* play roles in golgi membrane functions. In fact, *FAM198B* was expressed in mammary gland tissues and also significantly correlated to CHL_fat concentration (Tables 5 and S4b), so its role in CHL synthesis in the mammary gland warrants further investigation.

An intergenic region of BTA 17, position 63 Mb, is another interesting region harboring two suggestive SNPs (ARS-BFGL-NGS-64029 [rs110842600] (p = 1.91E-04) and Hapmap52830-rs29014800 [rs29014800] (p = 5.80E-05)) for CHL_fat and CHL_milk, respectively (Table S1a,b). Among many genes (*PLBD2*, *SDS*, *RITA1*, *PTPN11*, *DTX1*, *RASAL1*, *LHX5*, *CFAP73*, *IQCD*, *DDX54*, *OAS2*, *TPCN1*, *SLC8B1*, *SDSL* and *RPH3A*) located within 0.5 Mb flanking regions of these two SNPs, protein tyrosine phosphatase 1β *(PTPN1)* has been directly linked to CHL metabolism^30–33^ and it has been identified as a candidate gene for both CHL_fat and CHL_milk in this study. Variants of *PTPN11* have been found to associate with serum CHL level in a sex-specific pattern in human^30^ while Lu *et al*.^32^ identified *PTPN11* as a candidate gene for human plasma HDL-CHL. In the mammary gland, *PTPN11* gene was moderately expressed and had tendency (p = 0.067) of being correlated to CHL_fat concentration (Table S4b), therefore more studies are required to validate its role in CHL metabolism.

The QTL region at 117.7 Mb of BTA 4 harboring suggestive SNP ARS-BFGL-NGS-20980 (rs110814823) (p = 4.26E-04) for CHL_fat also harbors several important genes of CHL metabolism such as 5-hydroxytryptamine (serotonin) receptor 5 A (*HTR5A)*^38,39^ and insulin induced gene 1 *(INSIG1)*^40,41^. *INSIG1* was the second most highly expressed gene among CHL_fat positional candidate genes in the mammary gland (Table S4), whereas *HTR5A* was not expressed in the mammary gland. However, the expression of *INSIG1* gene in the mammary gland was not significantly correlated to CHL_fat concentration. It was shown recently that downregulation of *INSIG1* gene in mammary gland tissues of lactating dairy cows following dietary supplementation with 5% linseed oil was predicted by Ingenuity Pathways Analysis software (Invitrogen, Carlsbad, CA, USA) to activate CHL concentration in the mammary gland^42^. Two flanking genes (disintegrin and metalloproteinase domain-containing protein 11 [*ADAM11]* and hexamethylene bisacetamide inducible 1 [*HEXIM1*]) of suggestive SNP ARS-BFGL-NGS-24479 (rs41916457) (p = 3.90E-04) at 45.1 Mb region of Bta 19 (Table S1a) have been reported to be involved in CHL metabolism^43–45^. However, the expression of both *ADAM11* and *HEXIM1* genes was not significantly correlated to CHL_fat concentration in this study.

The enrichment analyses identified several GO terms with protein kinase regulator activities including negative regulation of cyclin-dependent protein kinase activity (p = 0.001, most significant biological process GO term) and cyclin-dependent protein kinase regulator activity (p = 1E-04, most significant molecular function GO term). In fact, cyclin-dependent protein kinase has been identified as a key regulator of eukaryotic cell cycle^46^, and it might be linked to CHL metabolism via its role in the regulation of energy status^47,48^ or lipid metabolism in the liver^49^. Regulation of CHL homeostasis and CHL metabolism is associated with plasma membrane activities^50,51^. Enrichment results suggest a potential role of the (basolateral) plasma membrane in the regulation of CHL_fat. The plasma membrane was the GO term enriched with the largest number of positional candidate genes for CHL_fat while basolateral plasma membrane was the most significantly enriched cell component GO term for CHL_fat candidate genes (Tables 2 and S2a). Meanwhile, cell-cell signaling (p = 0.001) and cell communication (p = 0.004) (Table 2) were among the most significant biological processes GO terms for CHL_fat suggesting that the regulation of CHL_fat probably requires the interaction and shared signaling activities between different cell types. Among the five KEGG pathways significantly enriched for CHL_fat positional candidate genes, the tight junction pathway has important roles in the transportation of milk constituents in mammary gland cells^52,53^, therefore it might also function in the transportation of CHL from the blood stream into the mammary gland or from mammary gland cells (*de novo* synthesized) into milk. Focal adhesion is an important pathway for immune functions in bovine mammary cells^54^, for lactation involution^55^ and for epigenetic regulation of milk production^56^. The focal adhesion kinase protein has been found in bovine milk fat globule membrane which is the major store of CHL in milk^57^, therefore focal adhesion pathway might be important for milk CHL via its role in the milk fat globule. Many significant transcription factors enriched for CHL_fat positional candidate genes have multiple functions. For example, c-Myc (*MYC)* is essential for the regulation of cell cycle progression, apoptosis and cellular transformation^58,59^ while peroxisome proliferator activated receptor delta (*PPARD)* is important for the regulation of the transcription of genes associated with proliferation, metabolism, inflammation, and immunity^60^. In fact, *PPARD* is an important transcription factor regulating CHL metabolism since it plays important roles in the reverse CHL transport^61^.

For CHL_milk, the most significant SNP (ARS-BFGL-NGS-4939 [rs109421300]) is located in an intronic region of diacylglycerol O-acyltransferase 1 (*DGAT1*) gene at 1,801,116 bp on BTA 14. This SNP has been reported to be in complete linkage disequilibrium with the K232A substitution within the *DGAT1* gene in German cows^62^. This SNP is also important for milk fat^62^ and fatty acid components^63^. Moreover, we also reported high LD among SNPs within and around the *DGAT1* gene region (Fig. 2). Another significantly associated SNP for CHL_milk (ARS-BFGL-NGS-18365 or rs110892754) has been found to be important for 305 day milk fat yield^64^. The *DGAT1* gene and the centromeric region of BTA 14 is important for the regulation of milk traits (milk fat yield, fat%, protein yield and protein%)^62,64–69^. *DGAT1* is a key enzyme in triacylglycerol biosynthesis and also play important roles in the regulation of CHL metabolism^70–72^. In *ApoE* gene knock-out mice, *DGAT1* deficiency decreases CHL uptake and absorption^71^. Therefore, the significant SNPs detected for CHL content in this study suggests that the *DGAT1* gene and the centromeric region of BTA 14 might be important in the regulation of milk CHL content. In fact, the expression of *DGAT1* gene in mammary gland tissues was also significantly correlated to CHL_milk concentration (p = 0.011) (Table 6), suggesting that *DAGT1* might contribute to the regulation of CHL_milk metabolism in the mammary gland.

A significant SNP (ARS-BFGL-NGS-41837 or rs110597360) for CHL_milk on BTA 6 is located in an intergenic region and the nearest gene to this SNP is *ENSBTAG00000001751*, an orthologue of human CXXC finger protein 4 (*CXXC4)* gene. *CXXC4* encodes a CXXC-type zinc finger domain-containing protein that functions as an antagonist of the canonical wingless/integrated signaling pathway^73,74^. The role of this novel gene in CHL_milk is unknown. On BTA 15, Hapmap38637-BTA-88156 (rs41596665) was significantly associated with CHL_milk and its flanking gene, mastermind like transcriptional coactivator 2 (*MAML2*) encodes for a member of the mastermind-like family of proteins which play important roles in the Notch signaling pathway^75^. In fact, the Notch signaling pathway was one of the pathways enriched for CHL_milk positional candidate genes in this study and it has been shown to have important roles in mammary gland development^76^. The Notch signaling pathway is important in the regulation of cell fate, cell proliferation and cell death in development^77^; however, there is no report of its direct role in milk CHL metabolism. On BTA 17, Hapmap52830-rs29014800 (rs29014800) was significantly associated with CHL_milk (p = 1.58E-05) and also suggestively associated with CHL_fat (Tables 1 and S1a), therefore this SNP might be important in the regulation of milk CHL content. On BTA 18, Hapmap39330-BTA-42256 (rs41605812), located in an intronic region of cadherin 13 (*CDH13*) gene (Table 1), is important for CHL_milk. A SNP within *CDH13* has been reported to be associated with plasma adiponectin levels in Japanese population^78^ and with triglyceride/high density lipoprotein ratio in Korean cardiovascular patients^79^. This gene is moderately expressed in the bovine mammary gland and also showed a trend (p = 0.075) to correlate to CHL_milk concentration (Table S4c). However, the role of this gene in milk CHL metabolism remains to be characterized.

The enrichment results for positional candidate genes showed several GO terms related to heart development (Table 3) which might reflect the fact that many candidate genes for CHL also play roles in cardiovascular disease development or heart diseases. An interesting molecular function GO term enriched was interleukin-2 receptor binding. It is known that interleukin-2 gene plays important roles in the activation of *STAT5a* gene in mammary gland development^80^. Glycerolipid metabolism, another enriched pathway has been implicated in the biosynthesis of CHL^81,82^. Therefore, interleukin-2 receptor binding (GO term) and glycerolipid metabolism pathway might also play important roles in bovine milk CHL metabolism. Interestingly, the most important transcription factor enriched for CHL_milk candidate genes was liver X receptor (*LXR*) (p = 1.00E-11) which is an important regulator of CHL, fatty acid, and glucose homeostasis^83–85^. There are two LXR subtypes (*LXRα* and *LXRβ)* and *LXRα*, the dominant subtype is highly expressed in the liver and other tissues (intestine, adipose, kidney, and adrenals)^86^ while LXRβ is widely expressed in different tissues^86^. In our mammary gland RNA expression data, *LXRβ* (or *NR1H2* gene) was also expressed at a higher level when compared to LXRα (or *NR1H3* gene). In the liver, *LXRα* expression was not significantly correlated to CHL_milk during transition and early lactation^20^. Another notable transcription factor enriched for CHL_milk positional candidate genes was notch homolog 1 (*NOTCH1*) (p = 0.028) (Table 4), which indicates the importance of NOCTH signaling pathway in milk CHL regulation. The functions of highly interacted genes (*MAPK15*, *FAM83H*, *ARHGAP39*, *HEATR7A*, *CYHR1* and *CPSF1*) in CHL_milk protein interaction network (Fig. 4), as well as highly significantly correlated genes (*ENSBTAG00000048096*, *TONSL* and *ITGB1*) (Table 6) in CHL metabolism are unknown and warrant further investigation.

The genetic variants identified in this study may facilitate selection in commercial breeding schemes either by incorporation in marker-enhanced selection or via implementation of genomic prediction including these identified genetic variants in a customized SNP panel. However, it is also important to consider potential limitations of our study including the limited size of resource population for GWAS, the relaxed p-value threshold used to select SNPs for gene set enrichments, potential for false discovery errors for certain enriched gene ontologies and pathways with few enriched genes in the gene list. The results should be interpreted with caution since both the results of associations (GWAS) and correlations derived from RNA sequence data may not reflect actual causative relationships. As already mentioned above, most CHL in milk is derived from the diet (which is partly reflected as CHL concentration in the blood) while only a small proportion, about 20%, is synthesized *de novo* in the mammary gland. Therefore, association analysis considering data on both blood and milk CHL concentrations might enhance knowledge of the implicated candidate genes in the regulatory pathways of milk CHL concentration such as dietary CHL transport from blood to the mammary gland and *de novo* synthesis in the mammary gland. Moreover, integration of gene expression data from the mammary gland and other tissues like the liver could identify the link between the mechanisms regulating CHL in the mammary gland and other tissues, and how these connections influence *de novo* synthesis of CHL in the mammary gland and milk CHL concentration.

To the best of our knowledge, this is the first GWAS on bovine milk CHL. The strongest SNP associations with milk CHL were detected on BTA14 and BTA17. This study identified several candidate genes (*DGAT1*, *PTPN1*, *INSIG1*, *HEXIM1*, *SDS*, and *HTR5A*), also important for human plasma CHL and related traits, that might be important for bovine milk CHL. Novel candidate genes (*RXFP1*, *FAM198B*, *TMEM144*, *CXXC4*, *MAML2* and *CDH13)* for milk CHL content were identified. Enrichment analyses suggested the involvement of important gene ontology terms ((basolateral) plasma membrane and cell-cell signaling processes), pathways (tight junction, focal adhesion, Notch signaling and glycerolipid metabolism pathways), and several transcription factors (*PPARD*, *LXR* and *NOTCH1*) in the regulation of bovine milk CHL content. The expression of some positional candidate genes in the mammary gland and their correlation with milk CHL content was supported with RNA sequencing data and milk CHL concentrations from the same animals. This study has therefore provided an insight into the genomics of bovine milk CHL and identified potential candidate genes and pathways that might be further studied to identify/confirm casual mutations that might help in the selection of cows with desired milk CHL content.

## Materials and Methods

### Animal Resource and Cholesterol Measure

Animal selection and milk sampling has been described in our previous study^4^. In brief, 100 ml of milk from each of 1,848 cows from 29 herds (minimum: 33 cows/herd and maximum: 172 cows/herd) were used. The concentration of CHL in milk fat was determined by direct saponification and capillary gas chromatography according to Fletouris *et al*.^87^. About 0.2 mg milk fat was saponified in capped tubes with 0.5 M methanolic KOH solution by heating for 15 minutes and the unsaponifiable fraction was extracted with toluene and analyzed by capillary gas chromatography using Agilent HP 6890 Series Gas Chromatography (GC) System (Agilent Technologies, California, USA). The concentration C (mg/100 g of fat) of CHL (CHL_fat) in analyzed samples was calculated based on computed mass (nanograms) of the analyte in the injected extract. The concentration of CHL was expressed in mg/100 g of fat (CHL_fat) or mg/100 g of milk (CHL_milk). After editing data for cow registration number, dam and sire information, test date, parity and age at calving, a total of 1,793 cows with complete records were retained for further analysis.

### Genotyping and Genotype Quality Control

DNA was isolated from hair follicles of 1,200 (out of 1,848) cows and genotyped using the Illumina BovineSNP50K BeadChip following manufacturer’s instructions (Illumina Inc., San Diego, CA). Genotype quality control was implemented by discarding animals and SNPs with call rate <0.95 and SNPs deviating from Hardy Weinberg equilibrium (p < 0.0001). Missing genotypes were imputed with FImpute 2 software^88^ and subsequently SNPs with MAF <0.05 were excluded. After quality control, 40,196 SNPs and 1,183 animals were retained for the association analyses.

### Association Analyses

The association analyses were performed using a univariate single SNP mixed linear model implemented in DMU package^89^. In summary, the model for each SNP (analyzed individually) was as follows (model 1):

1 $$y=1\mu +XB+Za+mg+e$$

where *y* is the vector of phenotype (CHL_fat, CHL_milk), 1 is a vector of 1s with length equal to number of observations, *μ* is the general mean, X is an incidence matrix relating phenotypes to the corresponding fixed effects, and *B* is the vector for fixed effects which includes interaction between herd and parity and days in milk (DIM), *Z* is an incidence matrix relating phenotypes to the corresponding random polygenic effect, *a* is a vector of the random polygenic effect ∼N(0, A*σ*_*u*_^2^) (where A is the additive relationship matrix and *σ*_*u*_^2^ is the polygenic variance), *m* is a vector with genotypic indicators 2, 1, or 0 for genotypes AA, AB and BB, respectively associating records to the marker effect, *g* is a scalar of the associated additive effect of the SNP, and *e* is a vector of random environmental deviates: N(0, *σ*_*e*_^2^) (where σ_e_^2^ is the general error variance). The parameters of the model σ^2^_u_ and σ^2^_e_ were estimated using restricted maximum likelihood (REML) for each SNP. To determine the significantly associated SNPs, an F-test was used to test the null hypothesis H_0_: *β* = 0. Distribution of test statistics was assessed by quantile-quantile (q-q) plot generated from association tests and the deviation from the null hypothesis of no SNP association with the trait. The markers with *p* nominal < 5E-05 were considered genome wide significant^90^ and markers with *p* nominal from 5E-05 to 5E-04 were considered suggestively genome wide significant to avoid many false negative results caused by stringent Bonferroni correction.

### Detection of Linkage Disequilibrium Blocks

Since several significant SNPs may be clustered in the same region (QTL region), we performed Linkage Disequilibrium (LD) analysis to characterize Linkage Disequilibrium patterns (LD block) for these regions. The LD block was defined according to Gabriel *et al*.^91^ and was detected and visualized with Haploview software^92^. Gabriel *et al*.^91^ defined a LD block as a region within which 95% of SNP pairs show strong LD (strong LD is defined if the one-sided upper 95% confidence bound on D′ is >0.98 and the lower bound is above 0.7). Before constructing LD block, we excluded SNPs with call rate <0.95, SNPs deviating from Hardy Weinberg equilibrium (p < 0.0001) and SNPs with MAF <0.05 and Mendelian inheritance errors >1. During LD construction, pairwise comparisons of markers >500 kb apart were ignored according to default settings in the Haploview software.

### Gene Mapping, Pathways and Transcription Factor Enrichment

We selected both significant and suggestive SNPs for pathway analyses because assignment of genes using only genome wise significant SNPs may ignore potentially important SNPs with lower significant levels, consequently missing out on key putative candidates and associated pathways. Nearby genes within a flanking distance of 0.5 Mb from significant and suggestive SNPs were queried from Ensemble database (Ensembl 83, *Bos taurus* UMD3.1), using bedtools^93^. Genes were submitted to the Database for Annotation, Visualization, and Integrated Discovery (DAVID, http://david.abcc.ncifcrf.gov/) for KEGG pathways and Gene Ontology (GO) enrichment analyses^94^ while STRING v10.5^95^ database was used to assess protein-protein interactions. The human genome was selected as background for enrichment instead of the bovine genome in order to take advantage of a richer database of information on the genomics of human CHL. Annotated pathways and GO terms were tested for enrichment using Fisher exact test. Pathways/GO terms were declared significantly enriched if they did not appear by chance with *p* < 0.05^94^. For STRING^95^ enrichment, the default options were used with the network edge selected based on confidence level. The minimum confidence threshold was set-up at the medium level with score of 0.4. In addition, a comprehensive gene set enrichment analysis for transcriptional machinery using ChIP-X enrichment analysis (ChEA2015)^96^ was performed with Enrichr (http://amp.pharm.mssm.edu/Enrichr/)^97^. The transcription factors were declared significantly enriched at *p* < 0.05.

### Evaluation of Expression of Positional Candidate Genes Using Mammary Gland RNA-Seq Data

The RNA-Seq expression data of 12 cows used is a subset of the data from our previous study^42^. Cows were in mid lactation (day 120–180) and fed the control ration (Table S4a). The expression of positional candidate genes for milk CHL as read count (reads per kilo base per million mapped reads (RPKM)) is shown in Table S4b. The CHL content in milk obtained from the 12 cows on the same day that mammary gland biopsies where obtained for RNA-Seq was determined using the same methods described above^87^. The Pearson correlations of CHL content with the RPKM values of positional candidate genes were calculated using cor() function in R program^98^. The candidate genes were considered significantly correlated to milk CHL content at *p* < 0.05.

The care of animals and use procedures were according to the Canadian Council on Animal Care^99^ and were approved by the Animal Care and Ethics Committee of Agriculture and Agri-Food Canada.

## Electronic supplementary material

Supplementary Information

Table S1

Table S2

Table S3

Table S4

## Data Availability

The RNA sequence data has been submitted to the BioProject data base (BioProject ID: PRJNA301774) and it is available through this link: http://www.ncbi.nlm.nih.gov/bioproject/PRJNA301774).
