# Supplementary material for: Genome wide association study identifies novel potential candidate genes for bovine milk cholesterol content
Source: Sci Rep. 2018 Sep 5;8:13239. doi: 10.1038/s41598-018-31427-0 (PMC6125589; doi:10.1038/s41598-018-31427-0)
Supplement: Supplementary file 1 — Supplementary Information [file 41598_2018_31427_MOESM1_ESM.pdf]

## Supplementary Information

# Genome wide association study identifies novel potential candidate genes for bovine milk cholesterol content

Duy N. Do[1,2], Flavio S. Schenkel[3], Filippo Miglior[3,4], Xin Zhao[2*] and Eveline M. Ibeagha-Awemu[1*].

[1]Agriculture and Agri-Food Canada, Sherbrooke Research and Development Centre, Sherbrooke, QC, Canada J1M 0C8

[2]Department of Animal Science, McGill University, Ste-Anne-de-Bellevue, QC, Canada H9X 3V9

[3]Centre for Genetic Improvement of Livestock, Department of Animal Biosciences, University of Guelph, Guelph, ON, Canada N1G 2W1

[4]Canadian Dairy Network, Guelph, ON, Canada N1K 1E5

*Corresponding authors:

Xin Zhao: xin.zhao@mcgill.ca

Eveline M. Ibeagha-Awemu:Eveline.ibeagha-awemu@agr.gc.ca

**Table S1.** Single nucleotide polymorphisms (SNPs) significantly and suggestively associated with milk (a) CHL_fat and genes annotated within 0.5 Mb flanking regions of SNPs and, (b) CHL_milk and genes annotated within 0.5 Mb flanking regions of SNPs and (c) common annotated genes for both CHL_fat and CHL_milk.

**Table S2.** Enriched gene ontology and pathways for positional candidate genes of CHL_milk and CHL_fat

Table S3. Transcription factors enriched for positional candidate genes for (a) CHL_fat and (b) CHL_milk.

**Table S4.** Expression (read counts) of CHL_milk and CHL_fat positional candidate genes (a) and the Pearson correlation of cholesterol concentration in milk (CHL_milk) (b) and CHL in fat (CHL_fat) (c) with expression values of their position candidate genes.

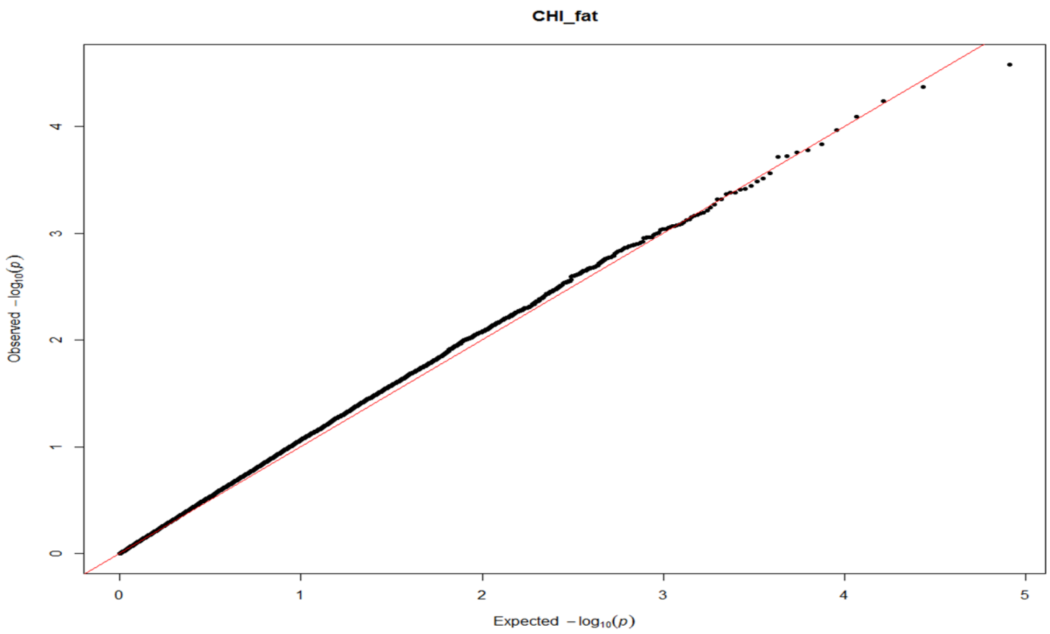


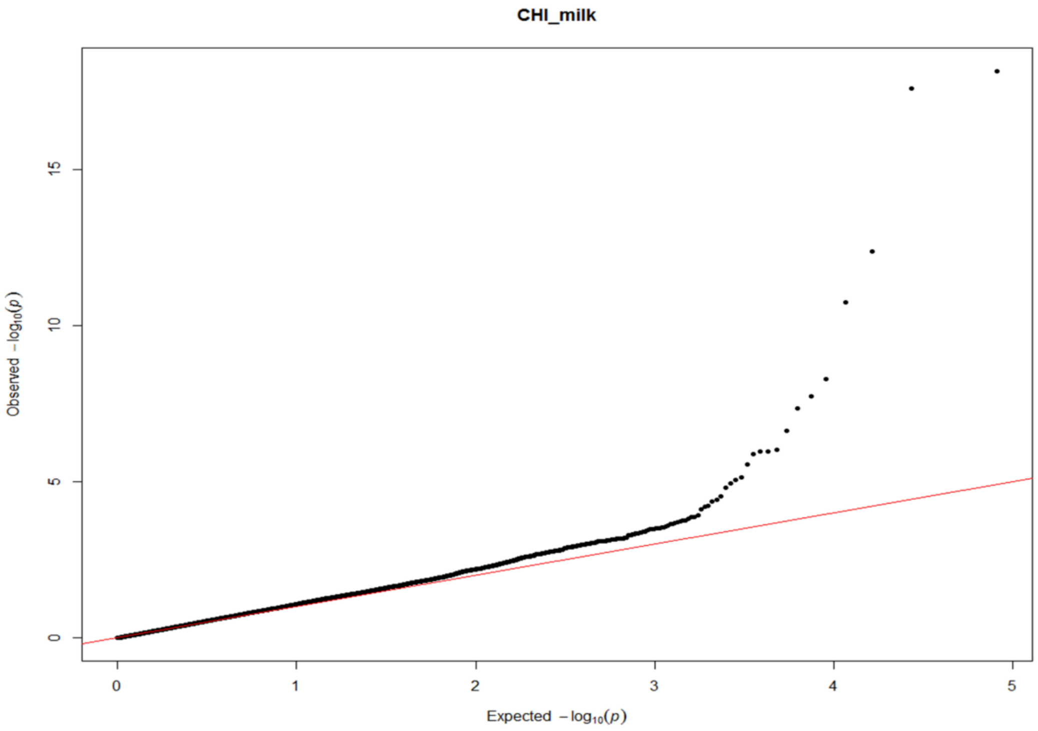


Figure S1. Quantile-quantile (qq) plots of observed plotted against expected p-values for CHL_fat and CHL_milk.
